# Pembrolizumab in advanced osteosarcoma: results of a single-arm, open-label, phase 2 trial

**DOI:** 10.1007/s00262-021-02876-w

**Published:** 2021-02-12

**Authors:** Kjetil Boye, Alessandra Longhi, Tormod Guren, Susanne Lorenz, Stine Næss, Michela Pierini, Ingeborg Taksdal, Ingvild Lobmaier, Marilena Cesari, Anna Paioli, Ayca M. Løndalen, Elisabetta Setola, Ivar Hompland, Leonardo A. Meza-Zepeda, Kirsten Sundby Hall, Emanuela Palmerini

**Affiliations:** 1 Department of Oncology, Oslo University Hospital, The Norwegian Radium Hospital, PO Box 4953, NO-0424 Oslo, Norway; 2 Department of Tumor Biology, The Norwegian Radium Hospital, Oslo University Hospital, Oslo, Norway; 3 IRCCS Istituto Ortopedico Rizzoli, Bologna, Italy; 4 Department of Core Facilities, The Norwegian Radium Hospital, Oslo University Hospital, Oslo, Norway; 5 Department of Radiology, The Norwegian Radium Hospital, Oslo University Hospital, Oslo, Norway; 6 Department of Pathology, The Norwegian Radium Hospital, Oslo University Hospital, Oslo, Norway; 7 Department of Nuclear Medicine, The Norwegian Radium Hospital, Oslo University Hospital, Oslo, Norway; 8 Department of Experimental, Diagnostic and Specialty Medicine, Alma Mater Studiorum, University of Bologna, Bologna, Italy

**Keywords:** Osteosarcoma, Pembrolizumab, PD-1 inhibitor, PD-L1 expression, NanoString

## Abstract

**Aim:**

To evaluate the activity and safety of the PD-1 antibody pembrolizumab in adult patients with advanced osteosarcoma.

**Material and methods:**

The study was a single-arm, open-label, phase 2 trial in patients with unresectable, relapsed osteosarcoma. The primary endpoint was clinical benefit rate (CBR) at 18 weeks of treatment, defined as complete response, partial response, or stable disease using RECIST v1.1. The trial had a Simon´s two-stage design, and ≥ 3 of 12 patients with clinical benefit in stage 1 were required to proceed to stage 2. The trial is registered with ClinicalTrials.gov, number NCT03013127. NanoString analysis was performed to explore tumor gene expression signatures and pathways.

**Results:**

Twelve patients were enrolled and received study treatment. No patients had clinical benefit at 18 weeks of treatment, and patient enrollment was stopped after completion of stage 1. Estimated median progression-free survival was 1.7 months (95% CI 1.2–2.2). At time of data cut-off, 11 patients were deceased due to osteosarcoma. Median overall survival was 6.6 months (95% CI 3.8–9.3). No treatment-related deaths or drug-related grade 3 or 4 adverse events were observed. PD-L1 expression was positive in one of 11 evaluable tumor samples, and the positive sample was from a patient with a mixed treatment response.

**Conclusion:**

In this phase 2 study in advanced osteosarcoma, pembrolizumab was well-tolerated but did not show clinically significant antitumor activity. Future trials with immunomodulatory agents in osteosarcoma should explore combination strategies in patients selected based on molecular profiles associated with response.

## Introduction

The prognosis for patients with recurrent osteosarcoma is poor [1–4]. Patients with a surgically resectable relapse may become long-term survivors, while recurrent, unresectable disease is almost always fatal [2]. Second-line systemic treatment options include ifosfamide and etoposide with or without carboplatin, high-dose ifosfamide alone, or gemcitabine-based regimens [5, 6]. Tyrosine kinase inhibitors such as pazopanib, regorafenib, and cabozantinib also have significant anti-tumor activity [7–10] and represent alternatives to conventional chemotherapy. Still, systemic therapy in second and later lines seems to produce a limited prolongation in survival [11, 12], and new therapeutic approaches are needed.

Pembrolizumab is a highly selective humanized monoclonal antibody designed to directly block the interaction between the immune checkpoint programmed cell death protein 1 (PD-1) and its ligands PD-L1 and PD-L2, thereby enhancing anticancer T-cell activity. Pembrolizumab has shown durable antitumor activity in several solid tumor types and a favorable safety profile. The first study to systematically assess the activity of anti-PD-1-antibodies in sarcoma was the SARC028 trial [13]. In this phase 2 study, 22 osteosarcoma patients aged 12 years or older were included, of whom only one had an objective response. Further evidence on the lack of activity of immune checkpoint inhibition in osteosarcoma was recently provided from pediatric trials with pembrolizumab, nivolumab, and atezolizumab [14–16]. These three phase 1–2 trials had altogether 31 evaluable osteosarcoma patients, and none achieved a radiological response. In addition, a French study with metronomic cyclophosphamide and pembrolizumab reported partial response (PR) in one of 14 evaluable patients [17].

This study was designed to evaluate the antitumor activity and safety of pembrolizumab in adult patients with advanced osteosarcoma.

## Materials and methods

### Study design and participants

This study was a phase 2, single arm, open-label, interventional trial of pembrolizumab in patients with advanced osteosarcoma entitled “PROMO: A phase II study of Pembrolizumab in patients with Relapsed Or Metastatic Osteosarcoma not eligible for curative surgery.” Patients were eligible if they were aged 18 years or older, had histologically verified osteosarcoma of bone, had disease relapse or progression after at least one line of systemic treatment, were not eligible for curatively intended surgery, had an Eastern Cooperative Oncology Group performance status of 0 or 1, had adequate bone marrow function (absolute neutrophil count ≥ 1500 cells per μL, platelets ≥ 100 000 per μL, and hemoglobin ≥ 9 g/dL), renal function (creatinine ≤ 1.5 X upper limit of normal (ULN) or glomerular filtration rate ≥ 60 mL/min), hepatic function (total bilirubin ≤ 1.5 X ULN and aspartate transaminase and alanine transaminase ≤ 2.5 X ULN and albumin ≥ 25 g/L), and coagulation function (international normalized ratio ≤ 1.5 X ULN and activated partial thromboplastin time ≤ 1.5 X ULN). Patients were ineligible if they had active central nervous system metastases, had an additional malignancy that was progressing or required active treatment, had autoimmune disease that required systemic treatment in the past 2 years, or had had prior treatment targeting PD-1 or PD-L1. The trial was registered with ClinicalTrials.gov, number NCT03013127, prior to inclusion of the first subject. The protocol was approved by the appropriate institutional review board and ethics committee and was conducted in accordance with the Declaration of Helsinki. All patients provided written informed consent before enrolment.

### Procedures

Pembrolizumab 200 mg was administered as a 30 min intravenous infusion on day 1 and repeated every 21 days. Adverse events were graded according to the Common Terminology Criteria for Adverse Events (CTCAE) version 4.0. Imaging was performed using computed tomography (CT) scans every 6 weeks during treatment until week 18, and then every 9 weeks until progression of disease. Radiological response was assessed using the Response Evaluation Criteria in Solid Tumors (RECIST) v1.1. Optional 18F-fluorodeoxyglucose (FDG) positron emission tomography (PET)/CT imaging was scheduled at screening, week 6 and week 18. The tumor with the highest FDG uptake at baseline was used for measurement of maximum standardized uptake value (SUVmax). Metabolic response was evaluated with PET Response Criteria in Solid Tumors (PERCIST) v1.0 using SUVpeak based on body weight. Study biopsies were scheduled at screening and week 9. Patients were to complete EORTC QLQ-C30 questionnaires at screening and every 6 weeks during treatment.

### Outcomes

The primary endpoint was clinical benefit rate (CBR), defined as complete response (CR), PR or stable disease (SD) 18 weeks after start of treatment. Secondary endpoints included progression-free survival (PFS), overall survival (OS), overall response rate (ORR), duration of response, response rates assessed by 18F-FDG PET/CT, changes in health-related quality of life from baseline assessed using EORTC QLQ-C30, safety, and tolerability. PFS was measured from the date of first dose of pembrolizumab to the date of PD or death of any cause, whichever occurred first. Patients alive without PD at the time of their last study visit were censored at that date. OS was calculated from the date of first dose of pembrolizumab until date of death of any cause. Patients alive were censored at the time of their last survival follow-up visit. Data collection was locked as of April 15th 2020.

### Statistical analysis

The trial had a Simon’s two-stage design. We assumed that a CBR after 18 weeks of 20% is a level of activity that is not of interest for further clinical development, whereas a CBR of 40% is of clinical interest. The type I error used was 10%. The study had a power of 80% to reject the null hypothesis when the true CBR is 40%. Planned accrual for the first stage was 12 patients. If there were ≤ 2 patients with clinical benefit among these 12 patients, the study would be stopped. Otherwise, 13 additional patients were to be accrued for a total of 25. The null hypothesis would be rejected if ≥ 8 of the 25 fully evaluable patients derived clinical benefit. Survival was estimated using the Kaplan–Meier method.

### Immunohistochemistry

Sections of 3 μm were stained with hematoxylin and eosin and for PD-L1 expression. Immunohistochemical PD-L1 staining was performed using the Dako Omnis automated staining solution (Dako). Slides were pretreated using EnVision FLEX Target Retrieval Solution Low pH (Dako) and incubated with monoclonal anti-PD-L1 antibody (clone 22C3; Dako) for 40 min at 25 °C. PD-L1 expression was evaluated by a sarcoma pathologist (I.L.), and categorized as 0, 0–1, 1–10, 10–50, and > 50% positive membranous staining of tumor cells.

### RNA isolation and gene expression analysis

Total RNA from fresh frozen material was isolated using AllPrep DNA/RNA/miRNA Universal kit (Qiagen) following the manufacturer’s protocol. The RNA was quantified with NanoDropTM One (Thermo Fisher Scientific), and RNA integrity was measured with the Bioanalyzer 2100 (Agilent). Gene expression analysis was performed on the NanoString nCounterTM Sprint Profiler (NanoString Technologies) with 50 ng total RNA starting material for each sample. For each sample, two replicates were hybridized over night (16 h) to the Pan Cancer IO 360™ panel probe set. All twelve samples were further processed following the manufacturer’s protocol. Analysis of the data was performed using the nSolverTM software version 4 (NanoString Technologies) and applying normalization based on housekeeping genes showing low variance across all samples.

## Results

### Patients

Between May 31st, 2017, and Sept 27th, 2018, 12 patients were enrolled, six at Oslo University Hospital in Oslo, Norway and six at Rizzoli Orthopedic Institute in Bologna, Italy. Baseline patient characteristics are presented in Table 1. Median age was 43 years (range 19–55). Nine patients (75%) had primary tumors in the lower extremity, two in the jaw, and one in the sacrum. All patients had distant metastasis at time of inclusion, and four (33%) had metastatic disease at initial diagnosis. Median number of previous lines of chemotherapy was 3 (range 1–7).

**Table 1:** Baseline patient characteristics

	Number of patients (%)*
Age, median (range)	43 (19–55)
*Gender*	
Male	8 (67)
Female	4 (33)
*Primary tumor localization*	
Femur	5 (42)
Tibia	4 (33)
Other	3 (25)
*Histological subtype*	
Osteoblastic	5 (42)
Fibroblastic	3 (25)
Other	4 (33)
*Performance status*	
ECOG 0	9 (75)
ECOG 1	3 (25)
*Previous lines of chemotherapy*	
≤ 2	2 (17)
3	6 (50)
≥ 4	4 (33)

*Unless otherwise specified. *ECOG* Eastern Cooperative Oncology Group.

### Treatment and radiological response

**Fig. 1:**
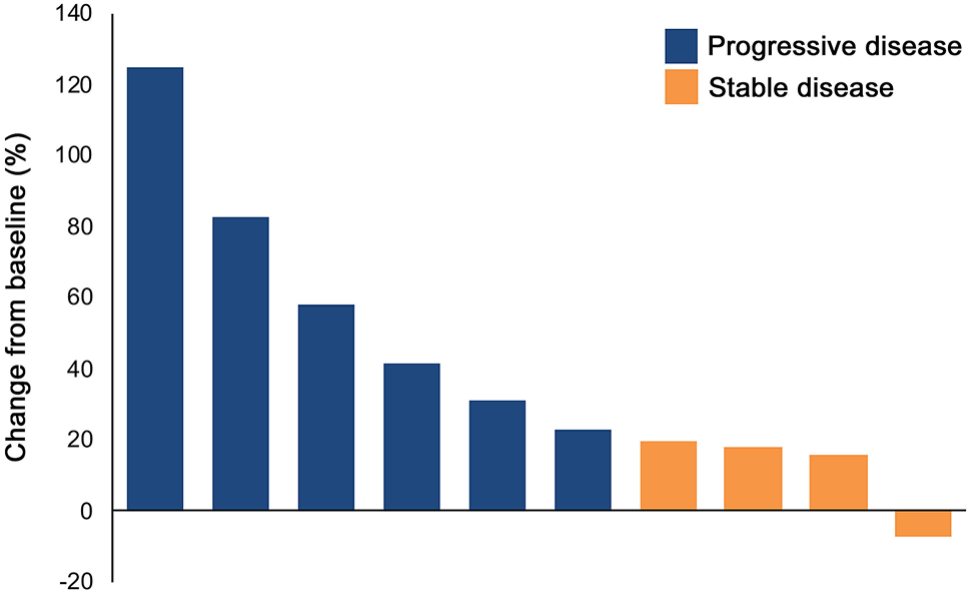
Waterfall plot showing best RECIST response. Individual patients are represented by vertical bars and the change in tumor size according to RECIST v1.1 is depicted on the Y-axis. Two patients did not undergo radiological evaluation and are not included

The median number of cycles of pembrolizumab administered was 2 (range 1–6). Four patients received only one cycle, all due to clinical progression prior to the second cycle. Ten patients underwent at least one radiological evaluation, while two patients with clinical progression were not evaluated radiologically. PD was observed in 9 of 10 patients, of whom six had PD at the first evaluation after 6 weeks and three at the second evaluation after 12 weeks. One patient had SD at first evaluation and stopped study treatment after cycle 3 because steroid therapy was initiated due to dyspnea and hemoptysis caused by a progressive lung metastasis, and was not subsequently evaluated. Thus, 0 of 12 patients reached the primary endpoint of clinical benefit defined as SD, PR, or CR at 18 weeks of treatment. Best overall response is shown in Fig. 1. Due to rapid disease progression, only four patients completed EORTC QLQ-C30 questionnaires during treatment, and quality of life results are thus not reported.

### Survival outcome

Nine patients (75%) had confirmed disease progression. Estimated median progression-free survival was 1.7 months (95% CI 1.2–2.2). At time of data cut-off, 11 patients were deceased, all from osteosarcoma, and estimated median overall survival was 6.6 months (95% CI 3.8–9.3).

### Adverse events

Adverse events of grade 3 or higher occurred in 7 of 12 patients (58%). Anemia grade 3 was reported in two patients, and increased alkaline phosphatase (grade 3), medullary compression (grade 3), pneumothorax (grade 3), and tumor-related pain (grade 3) in one patient each. No treatment-related deaths or drug-related grade 3 or 4 adverse reactions were observed.

### 18F-FDG PET/CT

Five patients underwent 18F-FDG PET/CT at baseline and after 6 weeks. The median SUVmax at baseline was 15.4 (range 4.1–20.5) and at first evaluation 11.4 (range 2.3–28.0). One patient had an increase in SUVmax from 20.5 to 28.0. SUVmax was reduced after 6 weeks in the other four patients, with an absolute reduction of 1.3–4.4 (12–44%) compared to baseline values. Three patients had progressive metabolic disease (PMD) at first evaluation using PERCIST v1.0. One patient with stable metabolic disease (SMD) at the first evaluation had PMD at the second PET/CT after 18 weeks, and one patient had SMD at both response evaluations.

### PD-L1 expression

**Fig. 2:**
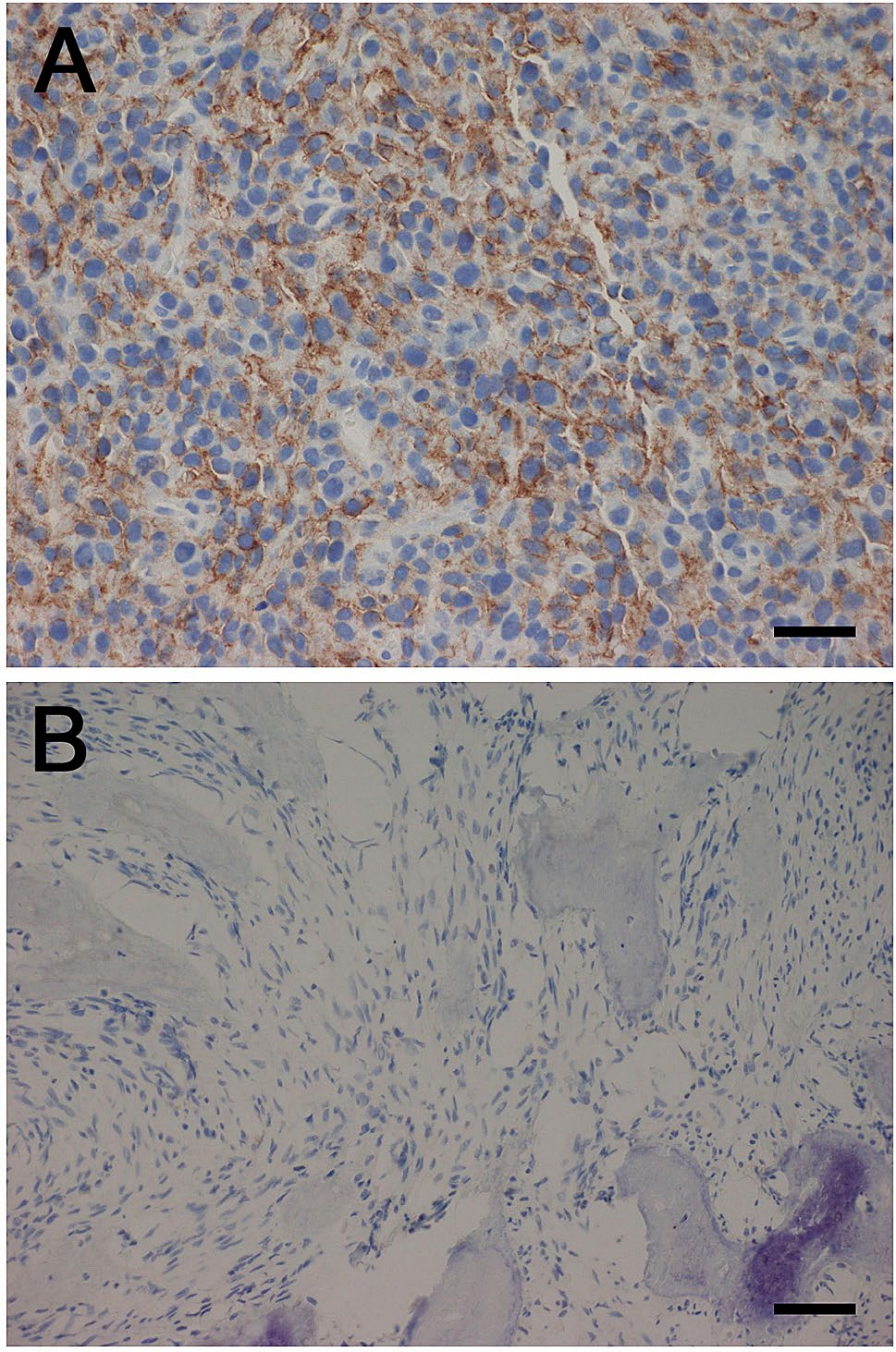
Photomicrographs of immunohistochemical staining with monoclonal anti-PD-L1 antibody (clone 22C3; Dako). **a** > 50% positive membranous staining of tumor cells. Scale bar 50 μm. **b** Negative staining. Scale bar 100 μm

**Fig. 3:**
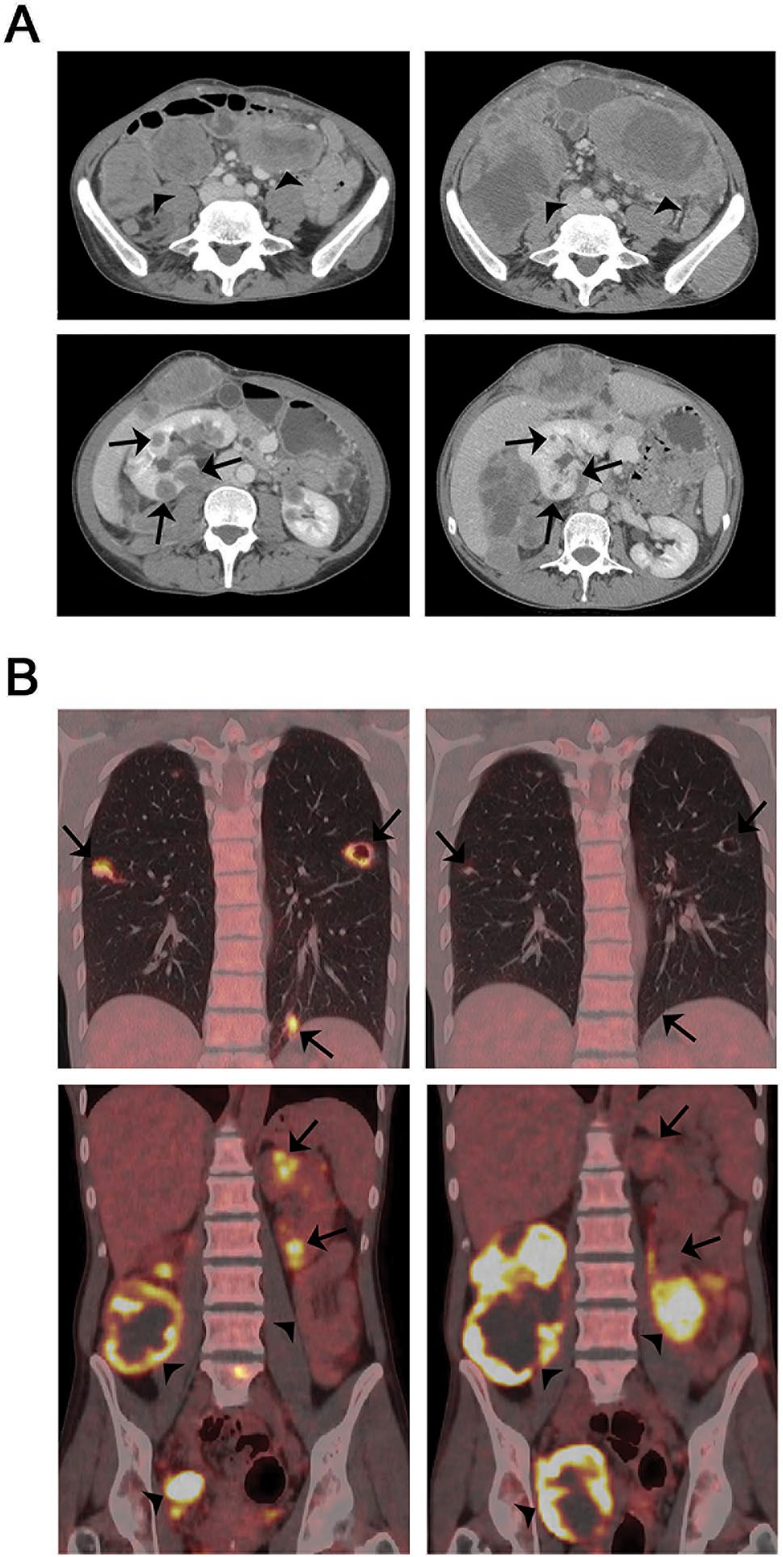
Baseline images and response evaluation after treatment with pembrolizumab in study patient 12 (PROMO-12). **a** CT images and **b** 18F-FDG PET/CT images at baseline (left panel) and after two cycles of pembrolizumab (right panel). Arrows indicate reduced uptake of 18F-FDG and tumor size in lung and kidney metastases, and arrowheads indicate progressive abdominal and pelvic metastases

Pretreatment tumor samples from 11 patients were available for analysis of PD-L1 expression. From three patients tumor tissue was obtained by a study-specific biopsy before treatment and from 8 patients archival tumor material was used for the analyses. In one sample, there was a strong positive membranous expression of PD-L1 (> 50% positive tumor cells), whereas the other ten samples were negative (Fig. 2). The PD-L1 positive specimen was a study biopsy of a soft tissue metastasis in the abdominal wall of a 41-year-old woman. She had PD and PMD after two treatment cycles, but with a mixed radiological and metabolic response. Uptake of 18F-FDG and tumor size were reduced in the lung and kidney metastases (Fig. 3, arrows), accompanied by an improved general condition and less tumor pain. There was, however, significant progression of abdominal and pelvic metastases (Fig. 3, arrowheads), and study treatment was discontinued.

### Gene expression analysis

**Fig. 4:**
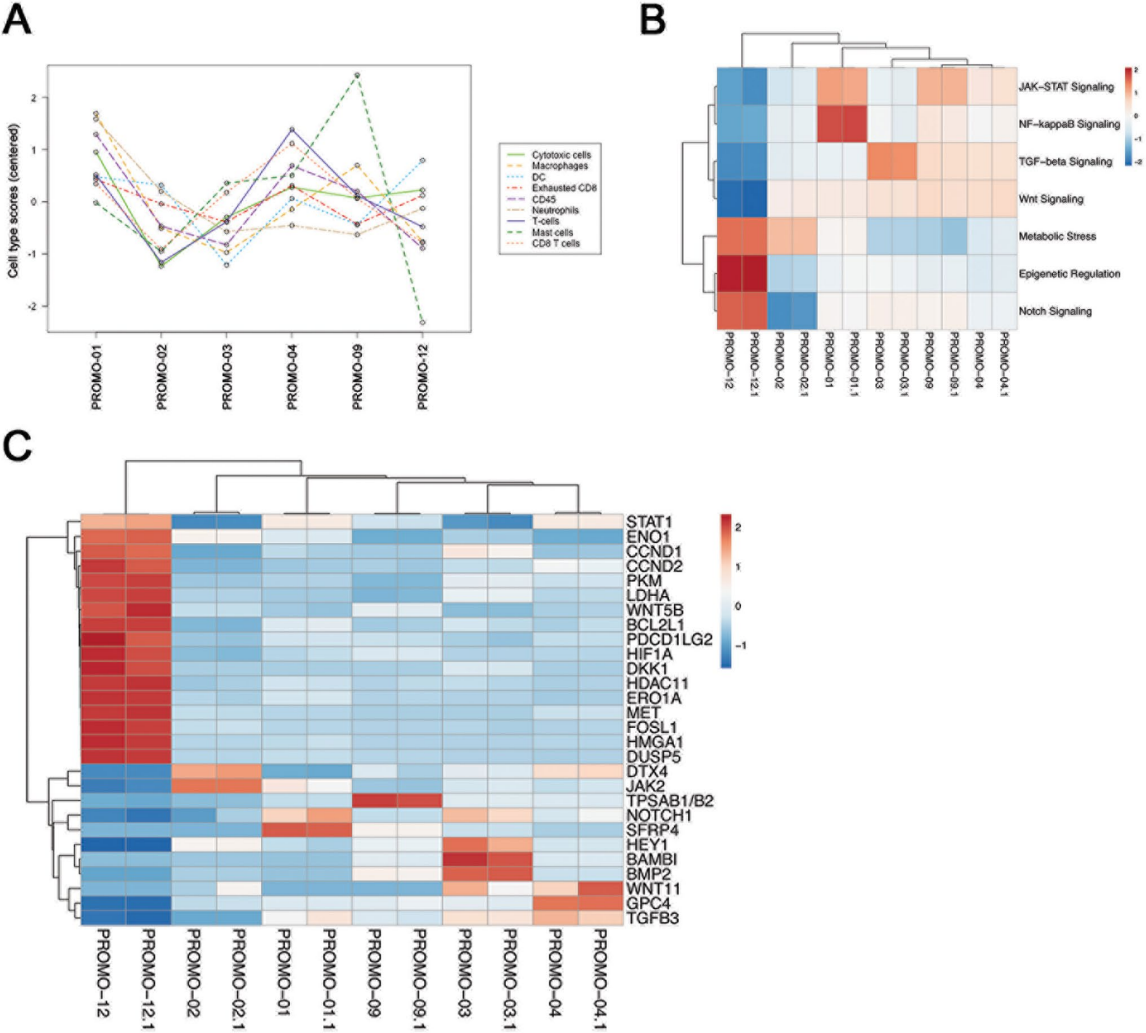
Gene expression analysis of tumor samples using the NanoString platform. **a** Abundance of various cell type populations across different samples. The cell type score is calculated based on cell-type specific gene expression markers. **b** Pathways showing differential behavior in PROMO-12 compared to the remaining samples. Pathway scores are calculated based on gene expression data using nSolver V4 software and oriented such that increasing score corresponds to increased expression. **c** Normalized gene expression values of top 28 differential expressed genes involved in these pathways. For both plots, rows are centered and unit variance scaling is applied to rows. Both rows and columns are clustered using correlation distance and average linkage. In **b** and **c**, samples were analyzed in duplicate and both duplicates are shown

To explore gene expression signatures and pathways, six available fresh frozen tissue samples were analyzed on the NanoString nCounter platform using the PanCancer IO 360™ Panel. We compared the gene expression from the patient sample with mixed response (PROMO-12) with the other samples. The overall significantly upregulated genes (*p* < 0.01) were *DUSP5*, *FOSL1*, *HMGA1*, *EROA1* and *MET*, and the significantly downregulated genes were *MAGEA3/A6* and *HEY1*. Quantification of cell populations based on gene expression profiles revealed that PROMO-12 had a lower infiltration of mast cells; otherwise, no clear differences in immune cell infiltration between the samples was observed (Fig. 4a). To search for pathways associated with antitumor activity of PD-1 inhibition, we compared signature scores from PROMO-12 with the remaining samples. PROMO-12 had lower activity in four pathways: JAK-STAT signaling, NF-kB signaling, transforming growth factor-β (TGF-β) signaling, and Wnt signaling (Fig. 4b). Three pathways showed higher activity: metabolic stress, epigenetic regulation, and Notch signaling (Fig. 4b). No clear differences were observed in the predicted activity of the other 18 pathways included in the analysis. Figure 4c shows the expression pattern of the most differentially expressed genes involved in the pathways with different activity levels in PROMO-12. Among these genes, we found the significantly upregulated genes *DUSP5*, *FOSL1*, *HMGA1*, *EROA1*, and *MET*, and further increased expression of *STAT1*, *CCND1*, *CCND2*, and *HIF1A*. In total, we detected 17 genes with increased expression and 11 genes with decreased expression, contributing to the pathway scores of the pathways with higher or lower activity in PROMO-12.

## Discussion

In this phase 2 study of adult patients with advanced osteosarcoma, pembrolizumab was well tolerated but did not show a clinically relevant antitumor activity. None of the 12 patients included in stage 1 of the trial achieved the primary endpoint of CR, PR, or SD at 18 weeks, and patient enrollment was thus stopped after completion of stage 1.

Our findings align well with other studies of single-agent immune checkpoint inhibitors in advanced osteosarcoma [13–17] and confirm that osteosarcomas generally show primary resistance to treatment targeting PD-1/PD-L1, both in adult and pediatric patient cohorts. Nevertheless, there is antitumor activity of PD-1/PD-L1 inhibitors in some osteosarcoma patients. One of our patients had reduced FDG uptake and reduced size of all lung and kidney metastases, accompanied by reduced symptoms, and one patient in the SARC028 study had a long-lasting partial response [13]. Molecular profiling of samples from such patients may reveal pathways or markers predictive of response, or provide information about therapeutic resistance. In our study, a strong PD-L1 expression was observed in a pretreatment biopsy from the patient with a mixed response, whereas all other samples were PD-L1 negative, in agreement with previous studies [18]. In SARC028, only three of 70 evaluable samples were PD-L1 positive, all of which were undifferentiated pleomorphic sarcomas [13]. Whether the responding osteosarcoma patient in that trial was among the evaluable samples was not specified. Although the overall predictive value of PD-L1 expression is uncertain in soft tissue sarcoma [19], the two patients with PD-L1 expression > 1% who were evaluable for response in the SARC028 trial had objective and durable responses. Furthermore, it is assumed that expression of PD-L1 is necessary, but perhaps not sufficient, for antitumor activity of immune checkpoint inhibitors targeting PD-1, and spatial and temporal variability in PD-L1 expression might explain response in tumors where no PD-L1 expression is detected in the examined tissue specimen. In PROMO-12, our patient with a mixed response, PD-L1 expression was strong both by immunohistochemistry and by mRNA expression analysis of a frozen specimen (data not shown), while all other investigated samples were negative.

TGF-β and Wnt signaling activity were reduced in PROMO-12 compared to the other analyzed samples (Fig. 4b). Tumor-intrinsic signaling through these pathways is associated with an immunosuppressive microenvironment and low T-cell infiltration [20, 21]. Thus, our findings might suggest that increased TGF-β and Wnt signaling associated with an immunosuppressive tumor microenvironment contributes to the lack of response to pembrolizumab in osteosarcoma.

Identifying mechanisms of primary resistance and strategies to overcome resistance should be a focus of further studies in osteosarcoma. Several mechanisms might explain the observed primary resistance, including lack of recognition by the adaptive immune system, insensitivity to the antiproliferative and proapoptotic effects of T-cell effector molecules, and an immunosuppressive microenvironment [22, 23]. A recent and comprehensive characterization of the immuno-genomic landscape of osteosarcoma suggests that the majority of osteosarcomas are “cold” tumors, with low levels of immune cell infiltration, low-to-moderate tumor mutation burden and lack of neoantigen expression [24]. Whole exome sequencing of four-matched tumor–normal pairs from our study was performed (data not shown), and confirmed a low tumor mutation burden with 6.6, 4.9, 5.3, and 2.6 single nucleotide variations (SNVs) per Mb. Combination strategies aimed at triggering immune cell activation and an immune-mediated antitumor response might be effective, such as the oncolytic virus talimogene laherparepvec that has entered clinical testing in soft tissue sarcoma [25].

In addition to combination strategies, enriching for patients who are more likely to benefit from immune checkpoint inhibition is probably necessary. Recently, it was shown that gene expression profiles associated with a high density of B cells and the presence of tertiary lymphoid structures predicted response to pembrolizumab in soft tissue sarcoma [26]. It will be of major interest to investigate whether these signatures are present in osteosarcoma samples, and if so, whether gene expression profiling could be used in future studies to select patients with a higher probability of response.

In conclusion, pembrolizumab was well-tolerated but had limited antitumor activity in adult patients with advanced osteosarcoma. None of the 12 patients in stage 1 of this phase 2 trial had clinical benefit, and patient enrollment was thus stopped. Future studies with immune checkpoint inhibitors in osteosarcoma should explore combination strategies in patients selected based on molecular profiles associated with response.
